# Experimental Infection of Squirrel Monkeys with Nipah Virus

**DOI:** 10.3201/eid1603.091346

**Published:** 2010-03

**Authors:** Philippe Marianneau, Vanessa Guillaume, K. Thong Wong, Munisamy Badmanathan, Ren Yih Looi, Séverine Murri, Philippe Loth, Noël Tordo, T. Fabian Wild, Branka Horvat, Hugues Contamin

**Affiliations:** Institut Pasteur, Lyon, France (P. Marianneau, S. Murri, P. Loth, N. Tordo, H. Contamin); Institut national de santé et de la recherché médicale, Lyon (V. Guillaume, T.F. Wild, B. Horvat); University of Malaya, Kuala Lumpur, Malaysia (K.T. Wong, M. Badmanathan, R.Y. Looi); 1 These authors contributed equally to this article.

**Keywords:** Nipah virus, emergent infection, primates, pathogenesis, squirrel monkey, *Saimiri sciureus*, ELISA, immunohistology, RT-PCR, encephalitis, viruses, zoonoses, dispatch

## Abstract

We infected squirrel monkeys (*Saimiri sciureus*) with Nipah virus to determine the monkeys’ suitability for use as primate models in preclinical testing of preventive and therapeutic treatments. Infection of squirrel monkeys through intravenous injection was followed by high death rates associated with acute neurologic and respiratory illness and viral RNA and antigen production.

Nipah virus (NiV) is a highly pathogenic zoonotic paramyxovirus that was first identified in Malaysia and Singapore in 1999 (*1*). Since the initial outbreak, NiV has been associated with human illness in Bangladesh and India (*2*) and was classified, together with the closely related Hendra virus, in the genus *Henipavirus*. Reported human death rates varied from 40%–92% (*3*), and some outbreaks were associated with human-to-human transmission (*4*). Most human infections led to encephalitis with vasculitis-induced thrombosis in the brain and atypical pneumonia in certain patients (*5*,*6*). Because of the lack of efficient treatment or a vaccine for Nipah virus and the high pathogenicity of the virus in humans, the manipulation of NiV requires BioSafety Level 4 (BSL-4) conditions.

Several species of fruit bats of the genus *Pteropus* are considered natural reservoirs of henipaviruses, although the disease does not develop in them (*7*). Pigs were responsible for amplifying the NiV infection in Malaysia, but their death rate was only 10%–15%. Laboratory infection of piglets caused development of neurologic signs in some animals, and NiV was detected in different tissues (*8*). Hamsters in laboratory studies are highly susceptible to NiV, and infection develops in multiple organs, including the brain (*9*). Cats infected with NiV in the laboratory reproduce the disease observed in naturally infected cats, including a severe respiratory and systemic disease, 6–13 days after infection (*10*). However, to our knowledge, a primate model necessary for preclinical testing of preventive and therapeutic approaches has not been described. We therefore assessed the squirrel monkey (*Saimiri sciureus*) as an experimental model of NiV infection.

## The Study

We selected these New World monkeys because of their availability, reliability as a primate model with which to study infectious diseases (*11*), and suitability as experimental animals in BSL-4 conditions. Thirteen 4-year-old male monkeys (0.8–1.0 kg) were imported from a breeding colony in French Guiana and housed in the BSL-4 animal care facility in Lyon. Experimental methods were approved by the Région Rhône Alpes ethics committee.

Twelve monkeys were infected with NiV isolate UMMC1 (*1*), GenBank accession no. AY029767, either intravenously or intranasally; for both modes of infection either 10^3^ or 10^7^ PFU was used. Animals were observed daily for 2 months for signs of disease onset; tissues were taken during the infection and at necropsy or at the end of experiment (Table 1). Blood samples were collected at different time points, serum samples were used for antibody analysis, and peripheral blood cells (PBMC) were used for RNA isolation. Different organ samples were taken and frozen at –80°C for RNA isolation or fixed in 4% formalin for histopathologic studies.

**Table 1:** Clinical course of Nipah virus infection in 12 squirrel monkeys*

Monkey	Mode of infection	Dose, PFU/mL	Day of 1st symptoms	Duration of clinical state	Day of euthanasia	Clinical state at euthanasia	Clinical signs
A†	IV	10^3^	–	–	3	Well	None
B	IV	10^3^	10	3	12†	Moribund	Uncoordinated motor movements, prostration and coma
C	IV	10^3^	19	3	21‡	Moribund	Uncoordinated motor movements, prostration, and coma
D†	IV	10^7^	–	–	3	Well	None
E	IV	10^7^	7	2	8†	Moribund	Uncoordinated motor movements, prostration, and coma
F	IV	10^7^	14	3	52	Recovered/well	Anorexia, depression
G†	IN	10^3^	–	–	4	Well	None
H	IN	10^3^	–	–	52	Well	None
I	IN	10^3^	8	3	56	Recovered/well	Anorexia, seizure
J†	IN	10^7^	–	–	4	Well	None
K	IN	10^7^			17		Septic shock not correlated with NiV infection
L	IN	10^7^	10	7	56	Recovered/well	Anorexia, seizure, edema of eyes

*IV, intravenous; IN, intranasal; NiV, Nipah virus. †Early systematic euthanasia. ‡Death caused by NiV infection.

RNA was extracted from different organs and analyzed by 1-step RT-PCR by using high fidelity PCR enzyme blend (Roche Applied Science, Mannheim, Germany) for NiV nucleoprotein expression as described (*12*). Detection of NiV-specific antibodies in the serum was performed simultaneously for all samples by ELISA and virus neutralization assays as described (*13*). Immunohistochemical analysis was conducted on formalin-fixed, paraffin-embedded tissues as described (*6*).

Onset of clinical illness was observed between 7 and 19 days postinfection (dpi), with development in the animals of anorexia, weight loss, and depression (characterized by slumped, collapsed body posture and lack of responsiveness to the environmental triggers). These clinical signs progressed for several hours and were associated with hyperthermia and an acute respiratory syndrome characterized by dyspnea and hyperventilation. During the course of the disease, the animals became more obtunded and had uncoordinated motor movements, ending, in some instances, with a loss of consciousness and coma (Table 1). Although clinical signs were seen in monkeys infected intranasally and intravenously, the disease lasted longer in intranasally infected animals (7 days) than in intravenously infected monkeys (2–3 days). With the latter, death was observed in 3 of 4 animals in which the disease was allowed to proceed. Clinical signs of illness for intranasally infected monkeys were milder and seen only in 2 of 4 animals before recovery after 3–7 days of illness. Clinical signs observed in monkeys appear to be similar to those reported for human infection, including involvement of neurologic and respiratory systems. In addition, the incubation period for the acute human infection in Malaysia was estimated to be from a few days to 2 weeks, total duration of illness ranged 2–34 days, and the rate of subclinical infection was ≈25% (*6*,*14*). It is possible that the inclusion of more animals in the study would have given higher heterogeneity in the course of disease, as seen in humans. Intravenous infection was much more efficient than the intranasal route in monkeys, probably because of a better delivery of the virus to different tissues.

NiV-specific RNA was detected in various organs only in intravenously infected animals (Table A1), demonstrating a differential virus spread, depending on time after infection and virus dose. Early detection after infection (3 dpi) was possible only in animal D, which was infected with a high dose of NiV. Animal F, which recovered from the disease, although positive for NiV (by RT-PCR) in the PBMC sample 2 dpi, was negative after necropsy on day 52, when virus was probably eliminated from the monkey. Detection of viral RNA in different tissues (liver, brain, spleen, kidney, lung, lymph nodes) early after infection suggests a rapid propagation of NiV and tropism for various tissues. Viral RNA was found in PBMC taken at different time points after infection, suggesting the role of these cells in viral propagation in the monkey. In contrast to what has been observed in hamsters (*9*), viral RNA was not detected in any urine samples from analyzed animals, thus excluding urine as a possible mode of virus dissemination in this species.

Monkeys showed mild histologic lesions, including the inflammation most obvious in the lung parenchyma (Figure, panel A). In contrast to human infection, vasculitis and brain abnormalities were much less evident. However, immunohistochemistry showed viral antigens immunolocalized to the brain, lung, spleen, and kidney extravascular parenchyma, thus confirming viral infection in these organs (Figure, panels B–E).

**Figure:**
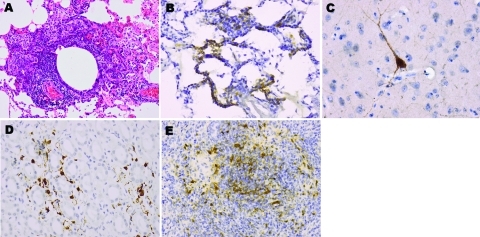
Pathologic signs associated with Nipah virus infection in squirrel monkeys. A) Focal inflammation in the lung (monkey B). Hematoxylin and eosin stains; original magnification ×10. B) Viral antigens (brown staining) were immunolocalized to the alveolar walls (monkey E). C) brain neuron (monkey B). D) Tubular and extratubular cells in the kidney (monkey E). E) Lymphoid cells in the spleen (monkey D). B–E, immunoperoxidase stains, original magnification ×20.

NiV-specific immunoglobulin (Ig) M responses were observed starting from 8 dpi for all monkeys except in groups H and I (Table 2). This finding suggests that 10^3^ PFU of NiV delivered intranasally was probably insufficient to induce infection in monkeys. Although NiV-specific antibodies were detected by ELISA in animals dying from the infection, sufficient titers of neutralizing antibodies did not develop in these monkeys and they were therefore not protected. These findings suggest the protective role of high neutralization titers in NiV infection. Our results agree with other studies of NiV infection that reported most human patients with fatal NiV infection had IgG and IgM in their serum and cerebrospinal fluid (*6*,*15*); neutralization titers were not analyzed in those studies.

**Table 2:** Detection of anti–Nipah virus antibodies in 12 squirrel monkeys by ELISA and seroneutralization assay*

Monkey	Mode of infection†	Serology‡	Day postinfection							
			2	3 or 4	8 or 9	12	17	30	37	52 or 56
A§	IV 10^3^	IgM	–	Neg						
	IV 10^3^	IgG	–	Neg						
	IV 10^3^	Neutralization	–	Neg						
B¶	IV 10^3^	IgM	Neg	–	Neg	0.396				
	IV 10^3^	IgG	Neg	–	Neg	Neg				
	IV 10^3^	Neutralization	Neg	–	Neg	Neg				
C¶	IV 10^3^	IgM	Neg	–	0.664	–	–			
	IV 10^3^	IgG	Neg	–	Neg	–	–			
	IV 10^3^	Neutralization	Neg	–	Neg	–	–			
D§	IV 10^7^	IgM	–	Neg						
	IV 10^7^	IgG	–	Neg						
	IV 10^7^	Neutralization	–	Neg						
E¶	IV 10^7^	IgM	Neg	–	1.343					
	IV 10^7^	IgG	Neg	–	0.562					
	IV 10^7^	Neutralization	Neg	–	40					
F	IV 10^7^	IgM	Neg	–	1.550	–	–	0.374	–	0.175
	IV 10^7^	IgG	Neg	–	0.369	–	–	2.867	–	3.023
	IV 10^7^	Neutralization	Neg	–	80	–	–	>1,280	–	>1,280
G§	IN 10^3^	IgM	Neg	Neg						
	IN 10^3^	IgG	Neg	Neg						
	IN 10^3^	Neutralization	Neg	Neg						
H	IN 10^3^	IgM	–	–	Neg	–	–	Neg	–	Neg
	IN 10^3^	IgG	–	–	Neg	–	–	Neg	–	Neg
	IN 10^3^	Neutralization	–	–	Neg	–	–	Neg	–	Neg
I	IN 10^3^	IgM	–	–	Neg	–	–	Neg	Neg	Neg
	IN 10^3^	IgG	–	–	Neg	–	–	Neg	Neg	Neg
	IN 10^3^	Neutralization	–	–	Neg	–	–	Neg	Neg	Neg
J§	IN 10^7^	IgM	–	Neg						
	IN 10^7^	IgG	–	Neg						
	IN 10^7^	Neutralization	–	Neg						
K	IN 10^7^	IgM	Neg	–	–	–	0.286			
	IN 10^7^	IgG	Neg	–	–	–	1.757			
	IN 10^7^	Neutralization	Neg	–	–	–	80			
L	IN 10^7^	IgM	–	–	0.375			0.248		Neg
	IN 10^7^	IgG	–	–	Neg			2.078		2.308
	IN 10^7^	Neutralization	–	–	Neg			320		320

*IV, intravenous; Ig, immunoglobulin; IN, intranasal; dpi, days postinfection. †Mode of infection and delivered viral dose (in PFU). ‡IgG and IgM antibodies were determined by ELISA and results are presented as absorbance readings. Neutralization titers are expressed as reciprocal values of 2-fold serum dilutions required to completely inhibit cytopathic effect of 25 PFU of Nipah virus on Vero cells. §Early systemic euthanasia: 3 dpi (A and D) or 4 dpi (G and J). ¶Death caused by Nipah virus infection, B: 12 dpi, C: 21 dpi, and E: 8 dpi.

Our results suggest some similarities of NiV pathogenesis in humans and squirrel monkeys, including development of clinical signs, progression of infection, and humoral immune response. We conclude that the squirrel monkey can be used as an animal model for experimental studies of NiV infection, and these results pave the way for further study.

## Appendix

**Table A1:** Detection of Nipah virus specific RNA by RT-PCR in organs from intravenously infected animals*

Infectious dose	10^3^ PFU				10^7^ PFU		
Animal number	A†	B‡	C‡	D†	E‡	F
Day euthanized	3	12	21	3	8	52
Source of sample							
Spleen	Neg	Pos	Neg		Pos	Pos	Neg
Liver	Neg	Pos	Pos		Neg	Pos	Neg
Lung	Neg	Pos	Neg		Pos	Pos	Neg
Heart	Neg	Pos	Neg		Neg	Neg	Neg
Intestine	Neg	Neg	Neg		Neg	Neg	Neg
Colon	Neg	Neg	Neg		Neg	Neg	Neg
Bladder	Neg	Pos	Neg		Neg	Neg	Neg
Kidney	Neg	Pos	–		Pos	Neg	Neg
Mesenteric lymph node	Neg	Pos	Neg		Neg	Pos	Neg
Auxiliary lymph node	Neg	Neg	Neg		Pos	Pos	Neg
Inguinal lymph node	Neg	Pos	Pos		Neg	Pos	Neg
Spinal cord	Neg	Neg	Neg		Neg	Pos	Neg
Brain (distal)	Neg	Pos	Neg		Neg	Pos	Neg
Brain (medium)	Neg	Neg	Pos		Neg	Neg	Neg
Brain (frontal)	Neg	Pos	Neg		Pos	Pos	Neg
Cerebellum	Neg	Pos	Neg		Neg	Neg	Neg
Urine	Neg	Neg	Neg		Neg	Neg	Neg
Plasma	–	Neg	Neg		Neg	Neg	Neg
PBMC (day of analysis§)	Neg	Pos (D9, 12)	Neg		Pos (D3)	Pos (D2, 8)	Pos (D2)

*Neg, negative; pos, positive; PBMC, peripheral blood mononuclear cell †Early systemic euthanasia. ‡Death caused by NiV infection. §Day postinfection when cells were obtained for the test.
